# Facilitators and barriers to behavior change in overweight and obesity management using the COM-B model

**DOI:** 10.3389/fpsyg.2024.1280071

**Published:** 2024-02-22

**Authors:** Vladimira Timkova, Daniela Minarikova, Lubomira Fabryova, Jana Buckova, Peter Minarik, Zuzana Katreniakova, Iveta Nagyova

**Affiliations:** ^1^Department of Social and Behavioural Medicine, Faculty of Medicine, Pavol Jozef (PJ) Safarik University in Kosice, Kosice, Slovakia; ^2^Department of Organisation and Management of Pharmacy, Faculty of Pharmacy, Comenius University in Bratislava, Bratislava, Slovakia; ^3^Biomedical Research Centre of the Slovak Academy of Sciences, Bratislava, Slovakia; ^4^Institute for Prevention and Intervention, St. Elisabeth University of Health and Social Work, Bratislava, Slovakia; ^5^Faculty of Nursing and Medical Professional Studies, Slovak Health University, Bratislava, Slovakia

**Keywords:** COM-B, obesity, overweight, weight management, behavior change

## Abstract

**Introduction:**

Increasing overweight and obesity rates represent one of the global public health challenges. COM-B is a theoretical model used to identify areas to target to achieve behavior change. It identifies three factors that are needed for any behavior to occur: capability, opportunity, and motivation. We aimed to assess the potential facilitators and barriers to behavior change in weight management using the COM-B.

**Methods:**

The study included 139 people with overweight and obesity (mean age 48.81 ± 14.49 years; 64.5% female; body mass index 32.64 ± 6.51 kg/m^2^; waist-to-height ratio 0.62 ± 0.10) from primary care settings. All participants completed the Brief Measure of Behavior Change (COM-B), the General Self-Efficacy Scale (GSE), the Rosenberg Self-esteem Scale (RSE), and the Overall Evaluation of Health (OEH). Multiple linear regression was performed to analyse the data.

**Results:**

The associations between sociodemographic and clinical variables and COM-B domains attenuated or were no longer significant when psychological resources were added to the regression models. Self-efficacy was identified as a stronger facilitator of health behavior change (*p* < 0.001) when compared to self-esteem (*p* < 0.05). No associations between automatic motivation and psychological resources were identified, however. Automatic motivation was found to be associated with higher age, being in a relationship, and better health.

**Discussion:**

Behavioral interventions for weight management should specifically target different components of COM-B. Self-efficacy and self-esteem may play a significant role in individual capabilities, opportunities, and reflective motivation and should be included in tailored public health interventions. Health programs targeting younger and single people, and people with chronic conditions may help to promote sustainable behavior change.

## Introduction

Obesity/overweight represents one of the global health problems in the 21st century. Based on the latest estimates, obesity affects nearly 25% of European adults, and combined overweight and obesity affects nearly 60% of all adults in Europe (World Obesity Federation, 2021; WHO Regional Office for Europe, 2022). By 2025 the prevalence of obesity in some EU countries may reach 30–43% (Pineda et al., 2018). Obesity/overweight rates have tripled in the past 30 years and continue to increase globally which is associated with a concomitant rise in health care and economic costs (Anekwe et al., 2020; WHO, 2021). Moreover, many obesity-related comorbidities lead to a decreased life expectancy (Anekwe et al., 2020).

Behavioral risk factors such as poor nutritional habits and physical inactivity cause 50% of all deaths in Slovakia, which is significantly above the EU average of 39% (OECD, 2019). Obesity rates in Slovakia are higher than the EU average (OECD, 2023), and obesity rates by education and income in Slovakia were also found to be larger than in the rest of the EU (OECD, 2019, 2021, 2023). Nevertheless, obesity as the second leading cause of preventable death does not receive prioritization in healthcare and public health interventions according to its prevalence and impact (De Lorenzo et al., 2020; World Obesity Federation, 2021). Thus, preventive strategies and effective weight management remain the most important challenges in public health (World Obesity Federation, 2021).

Behavioral insights currently represent one of the WHO/Europe flagship initiatives to improve the population's health (WHO, 2022). Current studies show that although caloric restriction-based interventions are effective in promoting weight reduction, long-term adherence to behavioral change should be supported (Hassapidou et al., 2023). Understanding human behavior can offer powerful insights to support health-protective changes in people's daily lives (WHO, 2022). Health behavior change interventions are crucial in obesity management, due to their focus on individuals' ability to self-regulate their health (Teixeira and Marques, 2017). Michie et al. (2014) proposed the “Capability, Opportunity, Motivation and Behaviour” (COM-B) as a theoretical model to guide understanding of behavior in different contexts, identify areas to target in order to enable behavior change and provide a basis for designing behavioral interventions in health management. Origins of COM-B can be found in health behavior models such as the theory of planned behavior, self-determination theory, transtheoretical model, or the health action process approach while meta-analyses show that these health behavior models can explain up to 37% of the variance in human behavior (Keyworth et al., 2020).

COM-B model defines behavior as the result of the interaction between the following components: physical and psychological capability (C), social and physical opportunity (O), and automatic and reflective motivation (M) (West and Michie, 2020).

It seems that health literacy alone is rarely sufficient to change an individual's behavior. Rather, psychological, social, and environmental factors, together with personal motivation (Kelly and Barker, 2016), or the effects of psychological factors on self-regulatory processes for overcoming lifestyle barriers should be considered (Annesi, 2020).

Thus, the understanding of COM-B domains allows for a more organized and efficient approach when considering barriers to health change in counseling people with overweight/obesity and how this knowledge can be applied in a healthcare setting (Sooknarine-Rajpatty et al., 2020). Application of the COM-B model considering core aspects of patient engagement is crucial to ensure the real impact of interventional programs can be achieved (Zheng et al., 2022). COM-B domains were also found to be predictive in long-term health-related behavior (Armitage and Munro, 2023) with current studies showing the explanatory potential and efficiency of COM-B components in the management of physical activity (Shoneye et al., 2020; Brierley et al., 2021; Kandel et al., 2021; Willmott et al., 2021; Niven et al., 2022; Gu et al., 2023; Huynh et al., 2023), and nutrition (Chater et al., 2020; Crowley et al., 2020; Shoneye et al., 2020; Kandel et al., 2021; Willmott et al., 2021) in the general population and people with overweight/obesity.

The study by Johnson et al. (2020) showed that self-efficacy and the COM-B domains are the key factors to allow personal tailoring of intervention techniques. Perceived self-efficacy facilitates goal-setting, effort investment, persistence in facing barriers, and recovery from setbacks (Schwarzer and Jerusalem, 1995). Psychological resources such as self-efficacy or self-esteem may also play a role in the process of making lifestyle changes (Wang and Veugelers, 2008; Dahlberg et al., 2022) and were identified as ideal targets for the maintenance of health behavior in high-income countries (Silveira et al., 2021). Low self-efficacy was found to be associated with sub-optimal self-care (Herber et al., 2017), and lower motivation and commitment to goals (Bandura, 1998), while low self-esteem was found to be associated with an inactive lifestyle (Wang and Veugelers, 2008). However, some current studies assessed the role of self-esteem and self-efficacy in weight management with inconclusive results (Olander et al., 2013; Jebeile et al., 2021; Björkman et al., 2022).

Health policies, services, and communication are frequently planned and administered without a thorough understanding of the drivers and barriers people face (WHO, 2022). Research on weight management omits factors that may affect human behavior (Ellis et al., 2019) and recent interventions focus mainly on dietary intake and a sedentary lifestyle although their success has been limited (Spinosa et al., 2019). Despite the overarching framework of the COM-B model to guide intervention development, there is a lack of quantitative studies that evaluate the mechanisms of action in COM-B (e.g., Keyworth et al., 2020) and its use and understanding in weight management is still scarce. Although information exists regarding specific behaviors associated with efficient weight management, far less is known about the role of psychological factors in determining if these health behaviors will be performed and maintained (Coms ´ a et al., 2023). Thus, our study aimed to assess the role of potential facilitators and barriers to weight-related behavior change using the COM-B model to identify and reflect on areas to target to achieve efficient weight management. We aimed to assess the association between sociodemographic variables, clinical variables, self-esteem, self-efficacy and COM-B domains in people with overweight and obesity.

## Materials and methods

### Participants

We included 139 people with overweight and obesity [64.5% females; 35.5% males; mean age 48.81 ± 14.49 years; body mass index (BMI) 32.64 ± 6.51 kg/m^2^; waist-to-height ratio (WHtR) 0.62 ± 0.10]. Participants were recruited by general practitioners (GPs) from outpatient clinical settings that covered all regions of Slovakia. The study sample consisted of consecutive patients who visited their GPs for routine medical examinations, preventive check-ups, or prescriptions of medication. The participants were included based on overall overweight and obesity (using BMI) and central obesity (using WHtR) scores. Exclusion criteria were a BMI of ≤ 24.99, and a WHtR of ≤ 0.49 which is not associated with central obesity or health risks (Ashwell and Gibson, 2016), the inability to speak the Slovak language, and being < 18 years of age. Additionally, people with major comorbidities such as cognitive impairment (e.g., due to dementia or stroke), current major psychiatric diagnoses (e.g., psychotic disorders, major depression), or diagnosis of terminal disease were also excluded by GP.

### Procedure

The study design was cross-sectional. Data collection consisted of self-reported questionnaires and measurements of patient height, weight, and waist circumference. Patients filled in the questionnaires at their own pace at home. Questionnaires were translated from the English language to the Slovak language following the standard procedure. First, two independent bilingual translators, one also an expert in psychology translated the questionnaires. Then, a forward-backwards translation was conducted. Differences between the original and the back translation were discussed by the research team in order to optimize the translation (International Test Commission, 2017). Informed consent was obtained from all individual participants included in the study. Participation in the study was entirely voluntary with no incentives offered for participation in the research. The study was approved by the Ethics Committee of Comenius University in Bratislava (approval no. EK 02/2020). All procedures performed in studies involving human participants were in accordance with the ethical standards of the institutional and/or national research committee and with the 1964 Helsinki Declaration and its later amendments or comparable ethical standards. Data were collected between October 2020 and August 2021.

### Measures

#### Self-efficacy

The 10-item General Self-efficacy Scale (GSE) reflects an optimistic self-belief (Schwarzer and Jerusalem, 1995) that one can perform novel, difficult tasks, or successfully cope with adversity in various domains of human functioning. The score ranges from 10 to 40 points and a higher score implies a higher level of internal-stable attributions of success (Schwarzer and Jerusalem, 1995). Cronbach's alpha in our sample was 0.93.

#### Self-esteem

The level of self-esteem was assessed using the Rosenberg Self-esteem Scale (RSE). RSE is a 10-item scale anchored at 4 points indicating a level of self-esteem. The total score ranges from 10 to 40 points, with a higher score indicating higher levels of self-esteem (Rosenberg, 1965). Cronbach's alpha in our sample was 0.85.

#### Capabilities, opportunities, and motivations

To assess barriers and facilitators in health-behavior change we used the Brief measure of capabilities, opportunities, and motivations (COM-B) which represents a valid questionnaire based on the COM-B model (Keyworth et al., 2020) that is used to estimate health-related behavior (e.g., Niven et al., 2022; Armitage et al., 2023). The COM-B model proposes that for someone to engage in a particular behavior at a given moment must have the physical and psychological capability, the social and physical opportunity to perform the behavior and, in addition, want or need to perform the behavior more than any other competing behaviors. Motivation includes all brain processes that guide behavior and covers basic drives and automatic processes such as habits, or impulses as well as reflective processes such as plans, intentions, or choices (West and Michie, 2020). The measure comprises six items designed to assess physical capability, psychological capability, physical opportunity, social opportunity, reflective motivation, and automatic motivation. Participants were asked to rate their ability and willingness to change their health-related behavior on an eleven-point scale (0 strongly disagree to 10 strongly agree). Health-related behavior change was defined as healthy nutrition and physical activity. Cronbach's alpha in our sample was 0.86.

#### Self-rated health

To assess the self-rated health the Overall Evaluation of Health (OEH) instrument was used. The OEH is a 100 mm visual analog scale on which the participant marks a line at the point that most closely reflects his/her self-rated health at present. The OEH score ranges from 0 to 100, with a higher score indicating better self-rated health (EURIDISS, 1990).

#### Sociodemographic and clinical variables

Sociodemographic data (age, sex, education, relationship status, household income) were collected using a questionnaire. Household income was categorized as low (lower than the “minimum wage”, i.e., under the poverty line), middle (at least “minimum wage” but less than double minimum wage), and high (twice the “minimum wage” and higher) income.^1^ Additional health information on clinical data such as the presence of chronic diseases was obtained from patients using a questionnaire. Data on weight, height, and waist circumference were assessed by healthcare professionals. The measures were taken barefoot with indoor clothes using an electronic scale for body weight, tape, and a wall-mount stadiometer. We calculated BMI in kg/m^2^ using weight and height measures. The waist-to-height ratio (WHtR) was used to assess central obesity. The WHtR of each participant was obtained by dividing waist circumference by height.

### Statistical analyses

Statistical analyses consisted of descriptive analyses of all key variables under study, followed by linear regression analysis (enter method) to assess the explained variance of COM-B domains. The order of variables included in the model was as follows: (1) age, sex, (2) education, income, relationship status, (3) presence of chronic disease, self-rated health, WHtR, (4) self-esteem, and (5) self-efficacy. Power analysis revealed that the statistical power for multivariate analysis exceeds 88% at α = 0.05 and medium effect size. The statistical power for bivariate analysis was 0.98 at α = 0.05 and medium effect size (Faul et al., 2009). A *p*-value of < 0.05 was considered to be statistically significant. All analyses were performed using the Statistical Package for the Social Sciences (IBM SPSS 26). Multicollinearity was assessed using the variance inflation factor (VIF < 2.5). A *p*-value of < 0.05 was considered statistically significant.

## Results

The basic description of the sample is shown in Table 1 (*N* = 139). The participants averaged 48.81 ± 14.49 years of age with the majority of females (64.5%) with a mean BMI of 32.64 ± 6.5 and WHtR of 0.62 ± 0.10. A total of 56.8% of participants had overall obesity and 54% were at severe risk of weight-related health problems. A total of 64.2% of participants suffered from chronic conditions with cardiovascular disease being the most prevalent (40.9%). The mean level of automatic motivation (5.75 ± 2.83) was lower than in other assessed COM-B domains.

**Table 1:** Characteristics of the study population (*N* = 139).

**Variables**	**Mean ±SD/%**
Age in years; mean ± SD	48.81 ± 14.49
**Sex (** ***N*** **;%)**	
Female	89 (64.5%)
Male	49 (35.5%)
**Education**	
Elementary	6 (4.4%)
Secondary	57 (41.6%)
University	74 (54.0%)
**Relationship status; single (** ***N*** **;%)**	**43 (31.2%)**
**Income (** ***N*** **;%)** ^†^	
Less than the minimum income^a^	6 (4.7%)
More than the minimum income^b^	26 (20.3%)
Double minimum income^c^	96 (75.0%)
**Body mass index (BMI); mean** ±**SD**	32.64 ± 6.5
Overweight (25.00–29.99)	60 (43.2%)
Obese (≥30.00)	79 (56.8%)
**Waist-to-height-ratio (WtHR)**	0.62 ± 0.10
WtHR with increased risk of health problems (0.50–0.59)	64 (46.0%)
WtHR with severe risk of health problems (≥0.60)	75 (54.0%)
**Presence of chronic disease (** ***N*** **;%)**	**88 (64.2%)**
**Number of chronic diseases (** ***N*** **;%)** ^††^	
One chronic disease	48 (54.5%)
Two comorbidities	27 (30.7%)
Three comorbidities	10 (11.4%)
More than three comorbidities	3 (3.4%)
**Type of chronic disease (** ***N*** **;%)** ^††^	
Cardiovascular diseases	36 (40.9%)
Diabetes	28 (31.8%)
Respiratory and pulmonary disorders	12 (13.6%)
Musculoskeletal disorders	28 (31.8%)
Sleep disorders	3 (3.4%)
Psychological disorders	13 (14.8%)
Endocrine disorders	21 (23.9%)
Gynecological disorders	2 (2.3%)
Neurological disorders	4 (4.5%)
Gastrointestinal disorders	3 (3.4%)
**Self-rated health (OEH) mean** ±**SD**	**60.10** ±**12.1**
**Self-esteem; mean, SD (RSE; 10–40)**	**31.01** ±**4.68**
**Self-efficacy (GSE; 10–40)**	**30.34** ±**5.76**
**COM-B questionnaire**	
COM-B social opportunities	7.55 ± 2.31
COM-B physical opportunities	7.10 ± 2.42
COM-B reflective motivation	7.39 ± 2.23
COM-B automatic motivation	5.75 ± 2.83
COM-B physical capabilities	7.12 ± 2.46
COM-B psychological capabilities	7.10 ± 2.38

^†^Household income per person per month.

^a^Less than 215 Euro per person/per month.

^b^215–429 Euro per person/per month.

^c^More than 429 Euro per person/per month.

^††^Number of chronic diseases and type of chronic condition was calculated out of the people with overweight/obesity who suffered from chronic disease (*N* = 88).

CD, chronic disease; OEH, self-rated health; RSE, Rosenberg Scale of Self-esteem, GSE, General Self-efficacy Scale; COM-B, brief measure of capabilities, opportunities, and motivations questionnaire; missing values: sex (0.7%); education: (1.4%); relationship status: (0.7%); income (7.9%); relationship status (6.2%); chronic disease: (1.4%).

Regression analyses showed that self-efficacy was a statistically significant contributor to overall explained variance in all final models except for the *Automatic Motivation* model. In this model, having a partner (β = 0.18; *p* < 0.05), and the presence of chronic disease (β = 0.24; *p* < 0.05), were significantly contributing to the overall explained variance. Higher age yielded a significant contribution also in the final model of *Automatic Motivation* (β = 0.21; *p* < 0.05) and *Psychological Capabilities* (β = 0.25; *p* < 0.01). Sex retained its significant contribution through each step of the analyses in the *Physical Capabilities* model (β = −0.18; *p* < 0.05 in the final iteration). Self-efficacy was most strongly associated with reflective motivation (β = 0.52; *p* < 0.001), followed by psychological capability (β = 0.44; *p* < 0.001) and physical capability (β = 0.43; *p* < 0.001). The associations between self-esteem, social opportunities, reflective motivation and physical capabilities were weaker but still significant (*p* < 0.05). Social opportunities and reflective motivation domains were associated with psychological factors, while sociodemographic and clinical variables were not significant. No significant associations between income, education, waist-to-height ratio and COM-B domains were identified. Detailed results of regression analyses are shown in Table 2.

**Table 2:** Regression analyses of six components of the COM-B regressed on sociodemographic, clinical, and psychological variables.

		**Physical opportunities**		**Social opportunities**		**Reflective motivation**		**Automatic motivation**		**Physical capabilities**		**Psychological capabilities**	
		*R* ^2^	**Beta**	*R* ^2^	**Beta**	*R* ^2^	**Beta**	*R* ^2^	**Beta**	*R* ^2^	**Beta**	*R* ^2^	**Beta**
Model 1	Age	0.01	0.15	−0.02	0.00	−0.01	0.07	0.01	0.16	0.03	0.06	0.07^**^	0.27^*^
	Sex		0.01		−0.02		0.05		0.01		−0.21^*^		−0.11
Model 2	Age	0.08^*^	0.13	0.06^*^	−0.01	−0.02	0.08	0.03	0.14	0.03	0.04	0.06^*^	0.26^*^
	Sex		0.06		0.05		0.05		0.01		−0.19^*^		−0.09
	Educ.		0.16		0.13		0.11		−0.03		0.03		0.05
	Income		0.05		0.19		−0.02		−0.03		0.03		0.05
	RS		0.22^*^		0.14		−0.04		0.21^*^		0.15		0.10
Model 3	Age	0.11^**^	0.13	0.09^*^	0.06	−0.01	0.10	0.12^**^	0.24^*^	0.14^**^	0.13	0.09^*^	0.31^**^
	Sex		0.05		0.02		0.05		−0.04		−0.23^*^		−0.12
	Educ.		0.17		0.14		0.12		−0.01		0.04		0.06
	Income		0.01		0.12		−0.07		−0.11		−0.09		−0.03
	RS		0.21^*^		0.16		−0.05		0.22^*^		0.16		0.10
	CD		0.09		0.15		0.08		0.28^**^		0.20^*^		0.12
	OEH		0.23^*^		0.06		0.18		0.08		0.22^*^		0.16
	WtHR		0.09		−0.12		0.01		−0.10		−0.13		−0.06
Model 4	Age	0.12^**^	0.09	0.13^**^	0.00	0.02	0.04	0.12^**^	0.21^*^	0.14^**^	0.13	0.11^**^	0.26^**^
	Sex		0.06		0.04		0.06		−0.03		−0.23^*^		−0.10
	Educ.		0.15		0.10		0.09		−0.02		0.04		0.04
	Income		−0.01		0.08		−0.11		−0.13		−0.09		−0.06
	RS		0.18		0.12		−0.09		0.20^*^		0.17		0.07
	CD		0.09		0.09		0.04		0.26^**^		0.20^*^		0.09
	OEH		0.18		−0.02		0.10		0.04		0.23^*^		0.09
	WtHR		0.09		−0.13		0.02		−0.10		−0.12		−0.06
	RSE		0.16		0.26^*^		0.25^*^		0.11		−0.03		0.20
Model 5	Age	0.17^***^	0.08	0.18^***^	−0.02	0.18^**^	0.02	0.13^**^	0.21^*^	0.24^***^	0.12	0.22^***^	0.25^**^
	Sex		0.10		0.07		0.12		−0.01		−0.18^*^		−0.05
	Educ.		0.11		0.05		0.01		−0.05		−0.02		−0.03
	Income		0.00		0.09		−0.09		−0.12		−0.08		−0.04
	RS		0.14		0.08		−0.15		0.18^*^		0.12		0.02
	CD		0.03		0.07		−0.01		0.24^*^		0.16		0.05
	OEH		0.16		−0.05		0.07		0.03		0.20^*^		0.06
	WtHR		0.11		−0.10		0.04		−0.09		−0.10		−0.04
	RSE		0.01		0.09		−0.01		0.03		−0.25^*^		−0.02
	GSE		0.30^**^		0.33^**^		0.52^***^		0.17		0.43^***^		0.44^***^

*R*^2^, adjusted *R* square; educ., education; RS, relationship status; CD, chronic disease; OEH, self-rated health; WtHR, waist/height ratio; RSE, Rosenberg Self-Esteem Scale; GSE, general self-efficacy; gender: a male was set as a reference; RS: being single was set as a reference; CD, having CD was set as a reference.

* *p* < 0.05;

** *p* < 0.01;

*** *p* < 0.001.

## Discussion

Physical capabilities, psychological capabilities, and automatic motivation domains were associated with higher age, female sex, having a partner, absence of chronic diseases, and better self-rated health. However, these associations between sociodemographic and clinical variables and COM-B domains attenuated or were no longer significant when psychological resources were added to the regression models. Self-efficacy was identified as a stronger facilitator of health behavior change when compared to self-esteem. Automatic motivation was found to be associated with higher age, being in a relationship, and better health. No association between automatic motivation and psychological resources was identified. Total explained variances in the final models varied from 13 to 24%.

Our study identified a significant association between all COM-B domains, except automatic motivation. These findings are in line with previous studies showing a significant association between self-efficacy, action orientation (Schwarzer and Jerusalem, 1995), reflective action control (Rothman et al., 2009), and motivation (Bandura, 1998). Furthermore, it is assumed, that as self-efficacy reflects beliefs about capabilities it may overlap with reflective motivation (e.g., Whittal et al., 2021). Previous research also identified self-efficacy as a mechanism of action by which interventions “work” in promoting behavior change (Werner et al., 2020). Therefore, the premise that self-efficacy may increase individuals' capabilities, opportunities, and reflective motivation can be used to develop intervention strategies.

We further found that the level of automatic motivation was the lowest in our sample when compared to other COM-B domains. Automatic motivation is needed to maintain health behavior change in the long term as it reflects habit strength (Gardner et al., 2022). However, previous studies also identified challenges in translating initial behavioral changes into long-standing habits (Rothman et al., 2009; Dunton et al., 2021). Thus, we may assume that people with overweight and obesity are more strongly motivated to health behavior change by conscious decision-making while their habit strength is weaker. Making behavior habitual, such that people automatically act in associated contexts due to learned context-response associations, offers a mechanism for maintaining new, desirable behaviors even when conscious motivation wanes (Gardner et al., 2022). It is therefore crucial to identify intervention strategies that would help to increase automatic motivation in people with overweight and obesity.

Success in long-term weight management depends partly on psychological factors (Oikarinen et al., 2023) and the importance of self-efficacy in predicting health intention and health behavior, is well-recognized (Sheeran et al., 2016; Zhang et al., 2019). We found no association between automatic motivation and intervention-prone psychological resources measured as self-esteem and self-efficacy, however. Previous studies showed that low self-efficacy was found to be associated with low motivation and lower commitment to goals (Bandura, 1998), low automaticity including poor intentional habit building (Stojanovic et al., 2021), and multiple concomitant eating behaviors that prevent successful weight management (Oikarinen et al., 2023). However, the lack of the association between self-efficacy and automatic motivation identified in our study is in line with previous findings that self-efficacy may be an important, but not sufficient condition for initiating long-lasting behavior change (e.g., Rothman et al., 2009). Moreover, people may overestimate their capabilities and only realize the difficulty in implementing lasting behavior change when it comes to real activity, also known as implementation dip in self-efficacy (Bandura and Locke, 2003).

Based on the results of our study it also seems that automatic motivation is lower in younger people with overweight/obesity. Older age may offer sufficient time to prevent unhealthy aging and to establish new daily routines to increase health behavior (Schoufour et al., 2021). As our data were collected during the COVID-19 pandemic we may also assume that older people might have been more worried about COVID-related complications (Barber and Kim, 2021) and thus been more strongly motivated to develop habitual health-related behaviors. However, current studies showed the opposite direction and identified younger people as more prone to lifestyle change during the pandemic (Barber and Kim, 2021; Oliveira et al., 2022). Thus, future studies are needed to shed more on the role of age in habitual change.

Based on our findings it also seems that in people affected by overweight and obesity, the presence of a chronic disease may play a more significant role in the low level of experienced automatic motivation, compared to psychological factors. The strength of automatic (e.g., habit-based) mechanisms was identified as a strong predictor of behavior change in people with chronic conditions and an intervention target for chronic illness self-management; while assessing it in practice settings may effectively detect poor to existing treatment and lifestyle regimens (Phillips et al., 2016). Diagnosis of a serious health condition should lead to the recognition of a problem, which represents an initial stage of change (Prochaska et al., 2005). Although we may assume that chronic disease may be a trigger for habitual health-related behavior change, previous studies found little evidence that severe chronic diseases such as cancer, diabetes, lung disease, or heart disease (Newsom et al., 2012; Williams et al., 2013) motivates health-protective changes and concluded that the vast majority of people newly diagnosed with a chronic condition do not adopt healthier behaviors (Newsom et al., 2012).

We also identified a significant association between automatic motivation and having a partner. The same was true for the association between relationship status and physical opportunities domain. No significant association between relationship status and psychological or physical capabilities, or the level of experienced reflective motivation, was identified. Thus, we may assume that having a partner seems to be positively associated with instrumental support—including physical opportunity (e.g., fewer time constraints, accessible childcare) (e.g., Ellis et al., 2019) and automatization of these processes in weight management, while emotional support of a partner seems to be negligible. It should be considered that the majority of our sample were females who globally do 75–83% of household chores and child or elderly care (The World Bank, 2018; Perez, 2019; Rubiano Matulevich and Viollaz, 2019) and thus may report having less leisure time when compared to males (Perez, 2019; Rubiano Matulevich and Viollaz, 2019). As the data were collected during the COVID-19 pandemic we may also assume that the increase in the gender gap in leisure time might have been highlighted (Yerkes et al., 2020; Clark et al., 2021) and people, especially women might have lacked opportunities for implementing sustainable weight management strategies with lack of instrumental support from the partner.

Based on our findings personalized interventions focusing on addressing poor health, younger people, and people without spouse/partner may help to promote behavior change via the automatization of motivation processes among adults with overweight and obesity. Our study suggests that intensive efforts are needed to maintain lifestyle improvements in this population.

We further found a lack of physical capabilities to make lifestyle changes to be associated with the presence of chronic disease, poor self-rated health, and low self-efficacy. We also found that the female sex was associated with lower physical capabilities to make lifestyle changes. These findings may be explained by poorer physical functioning reported by women compared to men identified in previous studies (Von Bonsdorff et al., 2011; Sialino et al., 2022), as well as barriers such as aspects of the built environment that may create gender gap in activity (Althoff et al., 2017) and decrease the functional capability of females (Satariano and Maus, 2017).

Surprisingly, an association between low self-esteem and higher physical capabilities reappeared when self-efficacy was added to the final model. This result may be explained by differences in sex, as physical capabilities are the only domain of the COM-B model that shows a significant contribution of sex to the overall explained variance in all stages of regression analyses. Adding self-efficacy to the model may point toward a closer association between self-efficacy and self-esteem in males compared to females, especially in the physical capabilities domain. These results need further investigation as a lower proportion of male participants may statistically impair these particular findings.

Better psychological capabilities were significantly associated with older age. Thus, it seems that lower age undermines psychological capabilities in people with overweight and obesity. However, this association was no longer significant when self-efficacy was added to the model. Self-efficacy was strongly associated also with psychological capabilities, while no association between self-esteem and psychological capabilities was observed.

No significant associations between education, income, waist-to-height ratio and COM-B domains were identified. As the difference in obesity rates by education and income in Slovakia are larger than in the rest of the EU (OECD, 2019, 2021), and the prevalence of behavioral risk factors was found to be higher among people with the lowest levels of education and income (OECD, 2023) more studies are needed to shed more light on the role of sociodemographic variables in overweight and obesity.

### Strengths and limitations

To our best knowledge, this is one of the first studies providing important insight into the factors enabling or inhibiting health behavior change to increase physical activity and healthy nutrition amongst adults with overweight and obesity using the COM-B model. To identify people with overweight and obesity, we used Body Mass Index (BMI) as one of the most widespread measures (e.g., Khanna et al., 2022) used in population studies (Garvey and Mechanick, 2020). However, it needs to be considered that the use of the BMI falls short in considering differences in body composition and the contribution of body fat to overall body weight (Pasco et al., 2014) and does not reflect differences in body composition according to the individual's age, sex (Gallagher et al., 1996), ethnicity-specific cutoffs (Caleyachetty et al., 2021), or obesity-related complications at the individual level (Garvey and Mechanick, 2020). Thus, we used also the WtHR of ≥0.5 as the main inclusion criterion for our study which led to the exclusion of three participants with BMI ≥ 25.0. Central obesity was also used as a control variable in multiple linear regression due to the collinearity between the waist-to-height ratio and BMI.

A brief measure of the COM-B questionnaire was previously used in the current studies that addressed health-related behavior (e.g., Niven et al., 2022; Armitage et al., 2023). The assessment of automatic motivation measured by COM-B is based on the Self-Report Habit Index, the most commonly used habit measure in health psychology, *which measures behavior XY as “something that a person does automatically*”. As the Self-Report Habit Index was found to be loaded on the same factor as did an instigation variant, whereas an execution variant was found to be loaded on a separate factor, there is also the possibility that we addressed habitual instigation, rather than its execution (Gardner et al., 2019). Thus, future research should be aimed at better understanding the contribution of automatic motivation to behavior frequency. As data were collected during the COVID-19 pandemic there is a possibility that our findings could be influenced by this global crisis and adopted measures. For example, physical opportunities, as well as social opportunities, might have been significantly limited by restrictions such as social distancing or lack of open public places (Porter et al., 2022). Although most of the current studies describe habitual processes including automatic motivation as a predictor of behavior change, the study by Spence et al. (2021) showed that physical opportunity and reflective motivation were the most consistent predictors of behavior change during COVID-19. Finally, as obesity/overweight tends to be higher among minority racial/ethnic groups (Dolinska et al., 2007), future research should aim to recruit an ethnically diverse sample of people with overweight/obesity.

### Implications for practice and future research

Obesity rates in Slovakia are on the rise and higher than the EU average. Low fruit and vegetable intake and insufficient physical activity of 30–40% of Slovak inhabitants represent another salient problem in health management (Maciejewski et al., 2021; World Obesity Federation, 2021; OECD, 2023). In addition, the health status of the Slovak population lags behind the EU average (OECD, 2019, 2021, 2023) while about half of the deaths in Slovakia can be associated with behavioral risk factors, including dietary risks and low levels of physical activity, which is higher than the EU average of two in five deaths (Maciejewski et al., 2021; OECD, 2021, 2023). Thus, it is crucial to identify the behavioral, social, and cultural factors that influence human behavior to provide more evidence-based, effective, and people-centered services and policies for effective health change (WHO, 2022).

From a clinical perspective, understanding patients' deficits or shortcomings, as well as resources and strengths may help to increase the self-care and management of the patient. Based on the results of our findings it seems that different types of interventions should specifically target different components of COM-B, with self-efficacy identified as the stronger facilitator of behavior change when compared to self-esteem. Self-efficacy may be improved using behavior change techniques such as goal setting and self-monitoring (Silveira et al., 2021). These findings can be implemented in interventional programs using Cognitive Behavioral Therapy or Acceptance and Commitment Therapy-based interventions that target people with overweight and obesity and may help to support efficient weight management and sustain regular engagement in behavioral change (Zhang et al., 2018; Cattivelli et al., 2021).

Although much remains to be done to equip interventionists with effective techniques for the enhancement of self-esteem or self-efficacy, it should be acknowledged that our findings confirm the assumptions that strong capabilities, opportunities, reflective motivation, and high self-efficacy may be an important, but not sufficient, condition for initiating behavior change as they are not always translated into action (e.g., Webb and Sheeran, 2006; Rothman et al., 2009). Thus, future research should shed more light on the possible deficits in automatic motivation, as well as the inability to manage the lack of habitual change through psychological interventions aimed at self-esteem and self-efficacy identified in our study.

As habit has been proposed as a means to promote the long-term maintenance of behavior (Gardner and Rebar, 2019), it needs to be considered that even when people successfully initiate the recommended changes, the gains are often transient as few of the traditional behavior change strategies have built-in mechanisms for maintenance (Gardner et al., 2012). Current studies using COM-B also show the necessity of supporting long-term adherence to behavior change in weight management (Hassapidou et al., 2023). Brief advice from a physician, nurse, or nutrition specialist on how to change, and engage automatic processes, may offer a valuable alternative with the potential for long-term impact and offers a useful option in the behavior change toolkit (Gardner et al., 2012). Based on our findings, to promote habitual change special attention should be paid to people affected by overweight and obesity of younger age, without a spouse or partner, and with chronic conditions. More research is needed on how automatic and reflective systems interact to promote or derail a positive health behavior change. Longitudinal studies on the changes in automatic motivation are needed as it is necessary to recognize that habit forms with multiple cue-behavioral response repetitions, and may follow a non-linear trajectory (Gardner et al., 2022).

## Conclusion

Behavioral interventions for weight management should specifically target different components of COM-B. Self-efficacy and self-esteem may play a significant role in individual capabilities, opportunities, and reflective motivation and should be included in tailored public health interventions. The results indicate deficits in automatic motivation, as well as the inability to manage the lack of habitual change through psychological interventions aimed at self-esteem and self-efficacy. It seems that health programs focusing on addressing poor health, younger people, and people without a partner or spouse may help to promote sustainable behavior change among people affected by overweight and obesity.

## Data availability statement

The raw data supporting the conclusions of this article will be made available by the authors, without undue reservation.

## Ethics statement

The studies involving humans were approved by Ethics Committee of Comenius University in Bratislava (approval no. EK 02/2020). The studies were conducted in accordance with the local legislation and institutional requirements. The participants provided their written informed consent to participate in this study.

## Author contributions

VT: Conceptualization, Data curation, Formal analysis, Funding acquisition, Investigation, Methodology, Resources, Writing—original draft. DM: Conceptualization, Writing—review & editing. LF: Data curation, Writing—review & editing. JB: Writing—review & editing. PM: Data curation, Writing—review & editing. ZK: Writing—review & editing, Conceptualization. IN: Conceptualization, Supervision, Writing—review & editing.

## Footnotes

^1^Ministry of Labour, Social Affairs and Family of the Slovak Republic (2022). Available online at: https://www.employment.gov.sk/en/family-social-assistance/material-need-assistance/subsistence-minimum.html.
